# Traumatic experiences, ICD‐11 PTSD, ICD‐11 complex PTSD, and the overlap with ICD‐10 diagnoses

**DOI:** 10.1111/acps.13161

**Published:** 2020-02-29

**Authors:** L. Møller, M. Augsburger, A. Elklit, U. Søgaard, E. Simonsen

**Affiliations:** ^1^ Psychiatric Research Unit, Region Zealand Slagelse Denmark; ^2^ Department of Clinical Medicine Faculty of Health and Medical Sciences University of Copenhagen Copenhagen Denmark; ^3^ Clinic for Traumatized Refugees, Region Zealand Slagelse Denmark; ^4^ Division of Psychopathology Department of Psychology University of Zurich Zurich Switzerland; ^5^ Department of Psychology National Centre of Psychotraumatology University of Southern Denmark Odense Denmark

**Keywords:** traumatic event, ICD‐10, ICD‐11, post‐traumatic stress disorder, complex post‐traumatic stress disorder

## Abstract

**Objectives:**

This study investigated the frequency of traumatic experiences, prevalence rates of ICD‐11 post‐traumatic stress disorder (PTSD) and complex PTSD (CPTSD), and overlap with ICD‐10 classified disorders in outpatient psychiatry.

**Method:**

Overall, 165 Danish psychiatric outpatients answered the International Trauma Questionnaire, the Life Event Checklist, and the World Health Organization Well‐being Index. ICD‐10 diagnoses were extracted from the hospital record. Chi‐square analysis, *t*‐tests, and conditional probability analysis were used for statistical analysis.

**Results:**

Nearly, all patients (94%) had experienced at least one traumatic event. CPTSD (36%) was more common than PTSD (8%) and had considerable overlap with ICD‐10 affective, anxiety, PTSD, personality, adjustment and stress‐reaction disorders, and behavioural and emotional disorders with onset usually occurring in childhood and adolescence. ICD‐11 PTSD overlapped with ICD‐10 anxiety, PTSD, adjustment and stress‐reaction disorders, and behavioural and emotional disorders with onset usually occurring in childhood and adolescence. A subgroup of patients with ICD‐10 PTSD (23%) did not meet criteria for ICD‐11 PTSD or CPTSD.

**Conclusion:**

Traumatic experiences are common. ICD‐11 CPTSD is a highly prevalent disorder in psychiatric outpatients. One quarter with ICD‐10 PTSD did not meet criteria for either ICD‐11 PTSD or CPTSD. PTSD and CPTSD had considerable overlap with ICD‐10 disorders.

**Significant outcomes**

- ICD‐11 CPTSD was more common (36%) compared with ICD‐11 PTSD (8%) in a psychiatric outpatient sample in Denmark.
- Comparison between ICD‐11 PTSD/CPTSD and ICD‐10 disorders showed profound overlap between both ICD‐11 PTSD/CPTSD and ICD‐10 disorders.
- About 23% with an ICD‐10 PTSD diagnosis did not meet the criteria for either ICD‐11 PTSD or CPTSD.

**Limitations**

- ICD‐10 diagnoses were extracted from hospital chart record and structural diagnostic interviews were not used.
- The ICD‐10 and ICD‐11 groups were relatively small, which may have reduced statistical power to detect true differences.
- We used self‐reported ICD‐11 diagnoses and were unable to check them with diagnostic interviews for potential response bias.

## Introduction

Exposure to traumatic experiences is found to be very high in psychiatric populations and multiple exposures are often the case (1, 2, 3, 4). Exposure to traumatic experience can lead to post‐traumatic stress disorder (PTSD); it is also a general risk factor for several mental disorders and may be associated with severity and comorbidity (5, 6, 7). This is especially true for interpersonal traumatic experiences that occurred in childhood (6, 8, 9, 10, 11). Exposure to specific types of traumatic experiences varies by sex, with men more frequently exposed to physical assault and combat and women to rape and sexual assault (12). However, studies show that women are more likely than men to develop PTSD. The reason for sex differences in PTSD is complex, with trauma type being just one possible explanation (13, 14, 15). Despite the evidence of high exposure to traumatic experiences in various psychiatric populations and its clinical consequences, exposure to traumatic experiences continues to be neglected in daily practice (3).

The 11th revision of the International Statistical Classification of Diseases and Related Health Problems (ICD‐11) has been officially released and includes a profound revision of trauma‐related disorders (16). The concept of PTSD is now described by a minimum set of symptoms that captures the core of post‐traumatic response. The aim was to minimize overlap with other diagnoses and enhance clinical utility. Complex PTSD (CPTSD) was included to capture broader and more complex post‐traumatic reactions (17). Diagnostic criteria for ICD‐11 PTSD include (i) re‐experiencing the traumatic experience in the here and now, (ii) avoidance of traumatic reminders, and (iii) heightened sense of current threat. CPTSD encompasses the PTSD diagnostic criteria and adds (iv) affect dysregulation, (v) negative self‐concept, and (vi) difficulties in relationships, characterized by disturbances in self‐organization (DSO). Both diagnoses require exposure to a traumatic experience and functional impairment. The diagnoses can be operationalized by the International Trauma Questionnaire (ITQ) (18).

The evidence base for the newly described ICD‐11 PTSD and CPTSD prevalence rates in clinical samples has (to the authors knowledge) mainly been derived from treatment‐seeking individuals in trauma specialty clinics and from refugee samples (18, 19, 20, 21). The prevalence rates have been found to range up to 61% for CPTSD and 25% for PTSD (18, 19), indicating that CPTSD is more common relative to PTSD in clinical samples. Whether the clinical picture of post‐traumatic response in psychiatric patients is captured by higher proportions of complex response is still uncertain. However, given that emerging evidence has found that exposure to traumatic experiences of interpersonal character, including unemployment and lower well‐being in childhood or adulthood (22, 23, 24, 25), is associated with CPTSD, it is highly likely that CPTSD is also highly prevalent in psychiatric patients.

Whether focusing PTSD symptoms on core post‐traumatic responses reduces overlap with other mental disorders, such as depression and anxiety, is still a topic for investigation. Few studies have investigated the overlap of ICD‐11 PTSD and CPTSD with other mental disorders. Three studies (26, 27, 28) comparing ICD‐11 and ICD‐10 PTSD found that individuals with ICD‐11 PTSD had equal or higher co‐occurrence with depression, anxiety, or somatic symptomatology than those who had ICD‐10 PTSD. However, these studies did not include ICD‐11 CPTSD for evaluation. Another study found high rates of comorbidity for both ICD‐11 PTSD and CPTSD in a trauma‐exposed community sample, although CPTSD was more strongly associated with major depressive disorders and generalized anxiety disorders than PTSD (24).

Evidence from studies using latent class analysis (18, 29, 30), network analysis (20), and confirmatory factor analysis (19, 31, 32, 33) have supported discriminant validity of the ICD‐11 PTSD and CPTSD symptom structure (34). Nevertheless, information from several studies comparing types of traumatic experiences between ICD‐11 PTSD and CPTSD yield different results. Multiple, chronic, and prolonged types of interpersonal traumatic experiences in both childhood and adulthood are found to be associated with greater likelihood for developing CPTSD than PTSD (24, 34, 35), but there is also evidence showing that a small proportion develop PTSD after such experiences (25, 34, 35, 36). CPTSD development has also been found in samples exposed to single‐incident traumatic experience (30). Females are more likely to have either of the two diagnoses than males (22, 24), and levels of well‐being are also found to differentiate CPTSD and PTSD, with CPTSD being associated with lower well‐being (22, 23). Patients in psychiatric facilities are among those with the highest exposure to traumatic experiences. Childhood interpersonal traumatic experiences are found to be a general risk factor for development of different psychiatric disorders, and low well‐being is associated with psychiatric morbidity. Whether these factors serve as differential risk factors between CPTSD and PTSD in a psychiatric population, where well‐being is low and exposure to multiple traumatic experiences are typically the case for every patient, is unknown.

Conditional prevalence rates concerning specific types of traumatic experience have so far only been studied once for ICD‐11 PTSD and CPTSD (37). This study found that sexual traumatic experiences, such as rape and sexual abuse, were both associated with a diagnosis of PTSD and CPTSD, and that prevalence of PTSD was also high after kidnapping and physical violence experiences. The findings were derived from a general population sample, and the conclusions were limited because of low reporting of specific event types leaving it to be further studied.

Little is known about how ICD‐11 PTSD and CPTSD unfold and differentiate in a psychiatric population. A study investigating these issues is important given that nearly all patients in psychiatric facilities are highly affected by traumatic experiences, and risk factors found to contribute to the development of ICD‐11 PTSD and CPTSD are also common. Psychiatric patients have high symptom pressure in general and therefore present a unique sample to test whether simplifying the definition of PTSD to core symptoms and including DSO symptoms to capture more complex responses reduces overlap with other diagnoses, and thus eases differential diagnostics.

### Study aims

Based on the limits in the previous literature, and considering that ICD‐11 will be the most widely used diagnostic manual in psychiatric facilities around the world, our specific aims were to: (i) determine frequencies and sex differences for traumatic experiences; (ii) investigate prevalence rates of ICD‐11 PTSD and CPTSD, their sex differences, and overlap with ICD‐10 diagnoses; (iii) provide conditional prevalence rates for ICD‐11 PTSD and CPTSD within the overall sample and the ICD‐10 PTSD group, given the experience of a specific type of traumatic event; and (iv) compare ICD‐11 PTSD and CPTSD on exposure to multiple traumatic experiences and psychological well‐being.

## Materials and methods

### Participants and procedures

Participants included a diverse sample of psychiatric patients recruited from three outpatient psychiatric clinics in the region of Zealand in Denmark. All patients consecutively referred for psychiatric treatment in a four‐month period (mid‐May to end of September, 2018) were asked to participate at their first or second clinical appointment. Patients who voluntarily agreed to participate (*n* = 195) were provided an iPad with a link to the online survey and completed the questionnaires in the waiting room or another suitable room where they would be undisturbed. On the iPad the patient was first presented with further study information together with a written consent form. No remuneration was given to the patients for participation. Ethical approval was granted by the science ethical committee in the region of Zealand. Confidentiality of the responses was secured through a unique link for each participant, which could not be reactivated after the browser was closed. The data were stored on an account that only the principle investigator could access.

Of the original 195 participants, 30 were excluded for non‐consent (*n* = 5) and incomplete survey data (*n* = 25). There was no significant difference in age between the excluded group (*M* = 33.5, SD = 14.03) and the final sample (*M* = 34, SD = 12.8), *t*(18.85) = 0.17, *P* = 0.87. There was also no significant difference in gender proportions between the excluded group (12 women, 71%; five men, 29%) and the final sample (123 women, 75%; 42 men, 25%), *P* > 0.05, Fisher's exact test. The final sample for analysis comprised 165 participants with complete data, mean age 34 (SD = 12.8, range 18–65). Seventy‐seven participants were single (47%), 27 were married (16%), 42 were living with a partner (26%) and 19 were living with others (12%). Half of the participants did not have children (*n* = 82), and 83 had one or more children. Most participants reported primary school as their highest education level (*n* = 62, 38%), followed by vocational education, bachelor degree or higher (both *n* = 35, 21%), and upper secondary school (*n* = 33, 20%). Further, 60 participants were on an illness leave (36%), 52 were employed or retired (32%), 42 were unemployed or in rehabilitation (both 26%), and 11 (7%) were receiving early retirement benefits.

### Measures

All measures were provided in Danish. If no formal translation existed, the measure was translated into Danish and professionally back‐translated. To ensure psychological terminology consistency in Danish, the final Danish version received consensus from a group of expert psychiatrists and psychologists.

### Traumatic experiences

The Life Event Checklist (LEC) (38) was used to assess for traumatic experiences. It consists of 16 items on traumatic experiences and an additional open‐response item about any other very stressful experience not listed. Three additional items were added to the list to specifically assess exposure before the age of 18: *childhood physical abuse (being hit, punched or hurt by someone responsible for caregiving such as a parent, foster parent, teacher, or coach)*, *childhood sexual abuse (being touched sexually or being sexually assaulted by someone older and a caregiver), and neglect (not being properly clothed or fed or being left without care)*. The items were coded as binary variables with endorsement of the traumatic experience as 1, otherwise 0. Total scores were calculated for self‐experienced events, with possible score ranging from 0 to 20. In addition, we estimated how many reported witnessing a close relative experience a traumatic event, coded 1 for item endorsement, otherwise 0.

### ICD‐11 PTSD and complex PTSD

To assess symptoms of ICD‐11 PTSD and CPTSD, we administered the preliminary version of the International Trauma Questionnaire (ITQ) with 28 items. Only the 18 items of the final and validated version were used in the analysis (18). We used endorsement to any of the LEC‐items instead of the ‘worst trauma’ item in the ITQ to identify whether the patient met the criteria of exposure to a traumatic experience. Responses to the self‐reported ‘worst trauma’ item were often unclear; for example, statements such as: ‘….’, ‘my anxiety’, or ‘life in general’ were frequent. We cross‐checked all participants who wrote about an experience that obviously did not qualify as extremely threatening or horrific by examining their LEC scores. All had endorsed one or multiple significantly horrific traumatic experiences. Six items measured symptoms of PTSD in the past month, with two items for each subdomain. Three items additionally assessed functional impairment levels related to PTSD. For CPTSD, additional DSO symptomatology was covered by six items with two items for each subdomain and three additional items for functional impairment related to the DSO domain. Participants were instructed to answer DSO domain questions in relation to how they typically feel, think about themselves, and relate to others.

The frequency of symptoms was assessed on a 5‐point Likert scale from 0 (‘not at all’) to 4 (‘extremely’). Diagnostic criteria required a score of 2 or higher (‘moderate’) for at least one of the symptoms in each cluster plus additional endorsement of two or higher (‘moderate’) for at least one functional impairment items. The diagnostic rules of ICD‐11 specify that a person can have either a PTSD or CPTSD diagnoses, not both at the same time. Thus, the scores for the ICD‐11 diagnoses were categorical.

The final ITQ psychometric properties have been shown to efficiently capture the distinction between PTSD and DSO symptomatology in clinical samples (18). Inter‐item correlation for PTSD and DSO domain and the entire ITQ scale were calculated to estimate internal consistency reliability because mean inter‐item correlations are not influenced by scale length and are more sensitive to multidimensional scales such as the ITQ (39). The average inter‐item correlations for DSO, PTSD, and the full scale were 0.45 (range: 0.32–0.51), 0.52 (range: 0.45–0.54), and 0.38 (range: 0.30–0.48), respectively, demonstrating acceptable values. The internal consistency measured with Cronbach’s alpha was also good (PTSD‐domain = 0.91 and DSO domain = 0.88).

### ICD‐10 diagnosis

The active ICD‐10 diagnoses for each participant were extracted from the hospital record after end data collection. The ICD‐10 diagnoses were based on a clinical assessment conducted during the first two to three appointments in the respective clinics. Since the ICD‐11 diagnoses were assessed at the first or second appointment in the clinic, no intervention was applied between the ICD‐10 and ICD‐11 diagnoses. The diagnoses were grouped in accordance with the categories in ICD‐10, chapter V. We made an exception for emotional unstable personality disorder of borderline type (BPD), adjustment and stress‐reaction disorders, and PTSD, which were given their own grouping variable because PTSD was an important variable for the analysis and adjustment, and stress‐reaction disorders have been placed under the same category in ICD‐11; there is debate regarding whether BPD can be differentiated from CPTSD (29, 34, 40). Our sample comprised the following diagnostic categories: PTSD (18%); anxiety, phobic, and OCD disorders (16%); emotional unstable personality disorder of borderline type (8%); other personality disorders (12%); adjustment and stress‐reaction disorders (11.5%); mood (affective) disorders (11%); behavioural and emotional disorders with onset usually occurring in childhood and adolescence (attention‐deficit hyperactivity disorders in Table 1) (8%); schizophrenia, schizotypal, and delusional disorders (2%); behavioural syndromes associated with physiological disturbances and physical factors (1.8%); mental and behavioural disorders related to psychoactive substance use (0.6%); and other disorders (18%). The other disorder category comprised individuals with code DZ038 *observation for other suspected diseases and conditions*, which is often given when the referral diagnosis is declined and further diagnostics need to be accomplished.

**Table 1:** Frequencies of traumatic experiences for total sample and sex

	Total (*n* = 165)	Sex		
		Female (*n* = 123)	Male (*n* = 42)	Gender difference
	*n* (%)	*n* (%)	*n* (%)	χ^2^ (1)
Natural disaster	14 (8)	10 (8)	4 (10)	n.s.
Fire or explosion	25 (15)	21 (17)	4 (10)	n.s.
Traffic accident	68 (41)	47 (38)	21 (50)	n.s.
Serious accident at work, home, or during recreational activity	31 (19)	21 (17)	10 (24)	n.s.
Exposure to toxic substance	11 (7)	5 (4)	6 (15)	n.s.
Childhood physical abuse	79 (48)	56 (46)	23 (55)	n.s.
Physical assault	90 (55)	58 (47)	32 (76)	10**
Assault with a weapon	39 (24)	22 (18)	17 (41)	8**
Sexual assault	41 (25)	38 (31)	3 (7)	8**
Childhood sexual abuse	32 (19)	27 (22)	5 (12)	n.s.
Other unwanted or uncomfortable sexual experience	56 (34)	51 (42)	5 (12)	10**
Combat or exposure to a war‐zone	4 (2)	2 (2)	2 (5)	n.s.
Captivity	13 (8)	10 (8)	3 (7)	n.s
Life‐threatening illness or injury	30 (18)	21 (17)	9 (22)	n.s.
Severe human suffering	34 (21)	23 (19)	11 (26)	n.s.
Sudden, violent death	20 (12)	16 (13)	4 (10)	n.s.
Sudden, unexpected death of someone close to you	84 (51)	62 (50)	22 (52)	n.s.
Serious injury, harm or death you caused to someone else	9 (5)	4 (3)	5 (12)	*
Any other stressful event or experience	43 (26)	33 (27)	10 (24)	n.s.
Neglect (before the age of 18)	64 (39)	51 (42)	13 (31)	n.s.

When no χ^2^ (df) is stated in the significance test Fisher's exact test was used. n.s. = *P* > 0.05; * = *P* < 0.05; ** = *P* < 0.01.

### Subjective psychological well‐being

We used the World Health Organization‐Five Well‐being Index (WHO‐5), a short, positively phrased 5‐item questionnaire that assesses subjective well‐being within the past two weeks. The items are scored on a 6‐point Likert scale ranging from 0 (‘*no times*’) to 5 (‘*all the time*’). For this study, the scores were summed and ranged from 0 to 25, where a raw score higher than 13 indicates psychiatric distress. In a review of 213 studies, WHO‐5 was established to have adequate reliability and validity for measuring well‐being (41). The inter‐item correlation for the WHO‐5 in the current sample was acceptable (0.47).

### Statistical analysis

All analyses were conducted in RStudio (version 1.2.1335). Frequencies and prevalence rates for traumatic experiences and ICD‐11 diagnoses were calculated. Statistical differences across ICD‐11 diagnoses and sex were tested via a series of Pearson chi‐square analyses, Fisher's exact tests, and *t*‐tests. Conditional probability analysis was calculated within the overall sample and in the group of ICD‐10 PTSD by the Bayes’ theorem formula for the probability that an individual would get either an ICD‐11 PTSD or CPTSD diagnosis given that the individual had prior exposure to a traumatic experience. ICD‐10 diagnoses with fewer than five participants were not independently tested because the small sample sizes would under power the analyses. Instead, they were grouped together and analyzed for overlap with ICD‐11 diagnoses.

## Results

### Traumatic experiences

Most of the participants (*n* = 155; 94%) had experienced at least one traumatic event, and the mean number of traumatic experiences was 4.78 (SD = 3.18; median = 4; range 0–15). One hundred ten participants (67%) had witnessed a close relative experience a traumatic event. The most common traumatic experiences were physical assault, sudden unexpected death of someone close, childhood physical abuse, and traffic accidents (Table 1).

Men experienced slightly more traumatic experiences (*M* = 4.93; SD = 3.30; median = 5; range = 0–15) than women (*M* = 4.73.; SD = 3.15; median = 4; range = 0–14), but the difference was not statistically significant; *t*(68.33) = 0.34, *P* = 0.74. However, significantly more men than women had experienced physical assault (χ^2^ (1) = 10, *P* = 0.002), assault with a weapon (χ^2^ (1) = 8, *P* = 0.006), and causing harm or death to someone else (*P* < 0.05, Fisher's exact test). Significantly, more women than men had experienced sexual assault (χ^2^ (1) = 8, *P* = 0.005) and other unwanted or uncomfortable sexual experiences (χ^2^ (1) = 10, *P* = 0.001).

### Prevalence rates of ICD‐11 PTSD/CPTSD, sex differences, and overlap with ICD‐10 diagnoses

The one‐month ICD‐11 prevalence rate was 8% (*n* = 13) for PTSD and 36% (*n* = 60) for CPTSD. There were no sex differences in PTSD (χ^2^ (1) = 2.1, *P* = 0.1), with 58% women (*n* = 7) and 46% men (*n* = 6) diagnosed with PTSD. We also found no sex difference in CPTSD diagnoses (χ^2^ (1) = 0.007, *P* = 0.93), with 73% of women (*n* = 44) and 27% of men (*n* = 16) diagnosed with CPTSD. Overlap between ICD‐11 PTSD/CPTSD diagnoses and ICD‐10 diagnoses are reported in Table 2. We only found statistically significant differences in ICD‐11 diagnoses on overlap with ICD‐10 adjustment and stress‐reaction diagnoses (*P* < 0.05, Fisher's exact test).

**Table 2:** Proportion of ICD‐11 diagnoses for total sample & ICD‐10 diagnoses

		ICD‐10 diagnoses							
	Total sample (*n* = 165)	Affective (*n* = 18)	Anxiety (*n* = 26)	PTSD (*n* = 30)	BPD (*n* = 13)	Other PD (*n* = 20)	Stress‐reaction (*n* = 19)	Attention‐deficit hyperactivity (*n* = 13)	Others (*n* = 26)
	*n* (%)	*n* (%)	*n* (%)	*n* (%)	*n* (%)	*n* (%)	*n* (%)	*n* (%)	*n* (%)
ICD‐11 diagnoses									
ICD‐11 PTSD	13 (8)	0 (…)	1 (8)	6 (46)	1 (8)	0 (…)	4 (31)	1 (8)	0 (…)
ICD‐11 CPTSD	60 (36)	4 (7)	8 (13)	17 (28)	6 (10)	5 (8)	5 (8)	7 (12)	8 (13)
ICD‐11 diagnoses differences		n.s	n.s.	n.s.	n.s.	n.s.	*	n.s.	n.s.

Fisher's exact test was used for testing significance. n.s. = *P* > 0.05; * = *P* < 0.05.

### Conditional probability of ICD‐11 PTSD and CPTSD

The highest conditional probability for ICD‐11 PTSD was found given that captivity (31%), combat or exposure to war (25%), severe human suffering (18%), and sexual assault (15%) had been experienced. The highest conditional probability for ICD‐11 CPTSD was found given that serious injury, harm or death you caused to someone else (89%), assault with a weapon (64%), and captivity (62%) had been experienced (Table 3).

**Table 3:** Conditional probabilities and odds ratio for type of traumatic experiences

	Total sample (*n* = 165)				ICD‐10 PTSD (*n* = 30)			
	PTSD	CPTSD	PTSD vs. CPTSD		PTSD	CPTSD	PTSD vs. CPTSD	
	n/C.pr.	n/C.pr.	χ^2^ (df)	OR (CI)	n/C.pr.	n/C.pr.	χ^2^ (df)	OR (CI)
Experienced potential trauma > 1	12/8%	59/42%	n.s.	4.76 (0.06 – 390.9)	6/20%	17/57%	n.s.	(…)
Natural disaster	0/ (…)	8/57%	n.s.	(…)	0/ (…)	5/83%	n.s.	(…)
Fire or explosion	1/4%	12/48%	n.s.	2.97 (0.37–138.69)	1/11%	5/56%	n.s.	0.49 (0.001–6.43)
Traffic accident	4/6%	28/41%	n.s.	1.95 (0.48–9.64)	1/6%	11/61%	n.s.	0.12 (0.02–1.43)
Serious accident at work, home, or during recreational activity	3/10%	15/48%	n.s.	1.11 (0.24–7.10)	1/10%	7/70%	n.s.	0.30 (0.01–3.63)
Exposure to toxic substance	1/9%	5/46%	n.s.	1.09 (0.11–55.84)	1/33%	2/68%	n.s.	1.47 (0.02–34.50)
Childhood physical abuse	6/8%	27/47%	n.s.	1.36 (0.33–5.68)	3/21%	9/64%	n.s.	0.89 (0.09–8.73)
Physical assault	9/10%	44/49%	n.s.	1.22 (0.24–5.17)	6/26%	14/61%	n.s.	(…)
Assault with a weapon	5/13%	25/64%	n.s.	1.14 (0.29–4.98)	4/22%	12/67%	n.s.	0.84 (0.08–12.15)
Sexual assault	6/15%	18/44%	n.s.	0.51 (0.12–2.09)	3/33%	5/56%	n.s.	2.3 (0.23–24.09)
Childhood sexual abuse	2/5%	17/53%	n.s.	2.15 (0.40–22.02)	2/22%	6/67%	n.s.	0.92 (0.06–8.95)
Other unwanted or uncomfortable sexual experience	3/5%	25/45%	n.s.	2.34 (0.53–14.67)	2/8%	7/64%	n.s.	0.72 (0.05–6.87)
Combat or exposure to a war‐zone	1/25%	2/50%	n.s.	0.42 (0.02–26.43)	1/100%	0/ (…)	n.s.	(…)
Captivity	8/31%	4/62%	n.s.	0.35 (0.07–1.94)	2/50%	2/50%	n.s.	3.50 (0.20–63.56)
Life‐threatening illness or injury	14/13%	4/47%	n.s.	0.69 (0.16–3.53)	1/13%	5/63%	n.s.	0.49 (0.01–6.44)
Severe human suffering	6/18%	16/47%	n.s.	0.43 (0.10–1.80)	3/27%	5/46%	n.s.	2.30 (0.23–24.09)
Sudden, violent death	2/10%	9/45%	n.s.	0.97 (0.16–10.47)	1/25%	2/50%	n.s.	1.47 (0.02–34.50)
Sudden, unexpected death of someone close to you	8/10%	41/49%	n.s.	1.34 (0.30–5.42)	3/19%	10/63%	n.s.	0.71 (0.07–6.97)
Serious injury, harm or death you caused to someone else	0/ (…)	8/89%	n.s.	(…)	0 (…)	1/50%	n.s.	(…)
Any other stressful event or experience	3/7%	24/56%	n.s.	2.20 (0.50–13.72)	1/11%	6/67%	n.s.	0.38 (0.01–4.74)
Neglect (before the age of 18)	6/10%	27/42%	n.s.	1.00 (0.24–3.89)	3/7%	9/87%	n.s	0.89 (0.09–8.73)

C.pr. = conditional probability; n.s. = *P* > 0.05; OR = odds ratio; CI = confidence interval; * = *P* < 0.05, **P < 0.01.

Fisher's exact test was used to test significant differences.

When we looked within the category of ICD‐10 PTSD we found the highest conditional probability for ICD‐11 PTSD, given exposure to combat or war‐zone activity (100%), captivity (50%) or toxic substances (33%). For ICD‐11 CPTSD, the highest conditional probability was found if the participants had experienced neglect (87%), natural disaster (83%), serious accident at work, home, or during recreational activity (70%).

### Comparison between ICD‐11 PTSD and CPTSD on exposure to multiple traumatic experiences and well‐being

We compared exposure to multiple traumatic experiences and well‐being for ICD‐11 PTSD and CPTSD diagnoses and found no significant difference in exposure to multiple traumatic experiences between ICD‐11 PTSD (*M* = 5.54, SD = 3.04) and CPTSD (*M* = 6.43, SD = 3.24); *t*(18.38) = −0.95, *P* = 0.35. We also did not find a significant difference in well‐being for ICD‐11 PTSD (*M* = 6.85, SD = 4.47) and CPTSD (*M* = 5.27, SD = 3.7); *t*(16.92) = 1.33, *P* = 0.20.

## Discussion

Our aim was to determine frequencies of traumatic experiences, investigate prevalence rates of ICD‐11 PTSD/CPTSD, and their overlap with different psychiatric disorders based on ICD‐10 classification in a psychiatric population to extend the previous research on traumatic experiences in psychiatric populations. It is one of the first studies to provide prevalence rates of ICD‐11 PTSD and CPTSD and their overlap with ICD‐10 disorders in a heterogeneous psychiatric population. Furthermore, it is the first systematic investigation of its kind in Denmark.

Irrespective of the psychiatric diagnoses, nearly all of the participants (94%) in this outpatient sample had been exposed to a traumatic experience. On average, the participants had lived through 4.5 different traumatic experiences and 67% had witnessed a close relative experience a traumatic event. In general, our findings are in line with previous research on psychiatric patients, finding that the majority of patients in psychiatry have been exposed to multiple traumatic experiences (1, 2, 3, 4). This indicates that trauma experiences are common in psychiatric outpatients in Denmark clinics.

In line with other studies (4, 12, 13), specific types of traumatic exposure in our sample seemed to follow sex‐specific patterns, with men more frequently exposed to violent physical assault and women to sexual assault. Nonetheless, we found comparable rates of number of traumatic exposures by sex. This contrasts with studies on the general population, where women tend to show slightly lower exposure to traumatic experiences (2, 12, 15). Even when traumatic experiences have been controlled for, most studies show that women are more likely to develop PTSD than men (13). In terms of ICD‐11 PTSD and CPTSD, there is inconsistency in the literature on whether sex can be conceived as a risk factor for developing either of the two diagnoses (24, 25, 42, 43). However, specifically for ICD‐11 PTSD, cumulative evidence suggests that being woman comes with an elevated risk even when controlling for exposure to specific trauma types (24, 25, 44). We did not find sex‐specific patterns in prevalence of ICD‐11 PTSD or CPTSD. Furthermore, we did not find significant differences on traumatic experiences that occurred before the age of 18, which some studies have found to heighten the risk for developing CPTSD (24, 25). The lack of sex‐specific patterns in prevalence of ICD‐11 PTSD and CPTSD could be the result of comparable high levels of traumatization experienced by the women and men in our study.

A main finding in our study was the considerably higher prevalence of ICD‐11 CPTSD diagnosis (36%) compared to PTSD (8%). This finding suggests that a high proportion of psychiatric patients reporting post‐traumatic responses justify a more complex symptom response and extends previous studies’ findings that populations with lower functioning and exposure to multiple traumatic experiences have a higher prevalence of CPTSD than PTSD (19, 44, 45, 46). The ICD‐11 PTSD/CPTSD diagnoses in our sample were spread across several ICD‐10 diagnoses. However, we did not find significant differences between ICD‐11 PTSD and CPTSD on overlap with ICD‐10 diagnoses, except for showing greater overlap between adjustment and stress‐reaction disorders and ICD‐11 PTSD (31%) than to ICD‐11 CPTSD (8%). Adjustment and stress‐reaction disorders and PTSD disorder are all causally related to a stressor. They can be distinguished by symptom intensity and time criterion duration. For adjustment disorder, the symptoms usually resolve within six months after the stressor and its consequences have ended. If the symptoms do not resolve, the diagnosis should be changed according to the clinical picture. Our findings indicate the general relationship between these two disorders. Additionally, a diverse range of traumatic experiences was reported by our study participants. As in previous studies (3, 24, 25), we found many people who suffered from a sudden loss of someone close (51%); thus, a mixture of both traumatic and grief‐inducing experiences were present in our sample. Traumatic loss may produce a sequelae of overlapping psychopathological symptoms and lead to different disorders, such as PTSD, depression, and anxiety (47). A recent study (47) investigated the co‐occurrence between prolonged grief disorder (PGD), PTSD, and adjustment disorder, and found that PTSD mediated the relationship between a serious life event, measured with summed LEC scores, and PGD. Increased life events affected PTSD, which then affected PGD. These findings broaden the potential traumatic experience consequences to include PGD; further so, given the findings of a significantly higher overlap between PTSD, adjustment and stress‐reaction disorders than CPTSD, adjustment and stress‐reaction disorders, adjustment and stress disorders may also contribute to a better understanding of how traumatic experiences impact the sequelae of stress‐related disorders.

For the remaining patients in our sample recognized as having ICD‐11 PTSD/CPTSD, patients diagnosed with an ICD‐10 affective disorder or personality disorder, other than BPD, were only found to overlap with ICD‐11 CPTSD. That we did not find overlap between ICD‐11 PTSD and ICD‐10 affective disorder or personality disorder, other than BPD, may be rooted in few participants in our sample meeting ICD‐11 PTSD criteria. However, it may also be a consequence of the narrower and more specific formulation of ICD‐11 PTSD. The direct comparison of ICD‐11 categorization across ICD‐10 diagnoses in our sample adds to earlier evidence (30, 48) that CPTSD overlaps with affective, personality, and anxiety disorders. We also found that CPTSD overlapped with behavioural and emotional disorders, with onset usually occurring in childhood and adolescence, and with adjustment and stress‐reaction disorders.

Barbano et al. (27) investigated differences in co‐occurring depressive and anxiety disorders in ICD‐10 and ICD‐11 PTSD diagnoses. In their sample of 3863 survivors of traumatic experiences, 51.3% who were identified with an ICD‐11 PTSD diagnosis also met criteria for a depressive disorder, and 20.3% met diagnostic criteria for an anxiety disorder. We did not find any overlap between ICD‐11 PTSD and ICD‐10 depressive disorders in our sample and only one patient with anxiety met criteria for ICD‐11 PTSD. As Barbano et al. (27) mentioned, they did not incorporate CPTSD in their evaluation of co‐occurrence. The participants who showed high co‐occurrence with depression and anxiety may have better fit a diagnosis of ICD‐11 CPTSD, than ICD‐11 PTSD. Another study on a combined sample of 399 survivors of either institutional abuse or war‐related childhood trauma by Glück et al. (26) found that the ICD‐11 PTSD formulation identified individuals that had more severe symptoms than the ICD‐10 PTSD formulation, and they also found that the formulation excluded individuals with milder PTSD. Their group of ICD‐11 PTSD reflected more severe cases and had equal comorbid conditions, such as depressive, anxiety, and somatic symptoms. A proportion of their sample may better be captured by an ICD‐11 CPTSD diagnose, which explains the equal rates of comorbidity. However, they did not include CPTSD as a measure. Individuals having ICD‐11 CPTSD are more likely to endorse symptoms of major depressive disorders and generalized anxiety than individuals having ICD‐11 PTSD (24).

Our findings that there is a tendency toward greater overlap between ICD‐11 CPTSD and ICD‐10 disorders should be interpreted with caution since we found no significant difference between ICD‐11 PTSD and CPTSD on overlap with most ICD‐10 diagnoses. How the DSO domain of CPTSD differs in psychopathological symptom profile from depression, anxiety, BPD, personality disorders, and behavioural and emotional disorders with onset usually occurring in childhood and adolescence should be the target of future studies to understand the divergent validity of ICD‐11 CPTSD in relation to these disorders.

A subset of seven patients (23%) with ICD‐10 PTSD did not meet the criteria for any of the ICD‐11 diagnoses assessed in this study. There are a few studies reporting reduced ICD‐11 PTSD prevalence rates relative to ICD‐10 PTSD rates (26, 28, 49, 50), with most finding an overlap between ICD‐11 and ICD‐10 PTSD, although a subset having ICD‐10 PTSD did not meet criteria for ICD‐11 PTSD. These studies estimated ICD‐11 PTSD diagnoses from different measures. Using ITQ, a specific measure for ICD‐11 PTSD/CPTSD, our results were similar to the earlier studies. Thus, it appears that ICD‐10 and ICD‐11 PTSD criteria capture different symptom patterns. A possible explanation may be found embedded in the goal to increase specificity of ICD‐11 PTSD by removing the symptoms that overlap between ICD‐10 PTSD and other disorders. Another possible explanation is the effect of a stricter definition of the re‐experience domain and the introduction of criteria that define functional impairment (17). The latter assumption is supported in the results from the study made by Glück et al. (26), which found the prevalence rate of PTSD to decrease from ICD‐10 to ICD‐11. This result was predominantly explained by the reduction in fulfillment of the re‐experience criteria. This is supported by comparable results on a combined sample of 345 U.S. military veterans and 2953 adults from the U.S. adult general population by Wisco et al. (28), who found a decrease in prevalence of ICD‐11 PTSD to be accounted for by changes in the re‐experiencing and hyperarousal domains. The above‐mentioned findings suggest that narrowing the definition of the re‐experiencing and hyperarousal domains may possibly account for the discrepancy in prevalence of PTSD diagnoses between the classification systems. What impact this alteration will have for individuals who have a clinically significant ICD‐10 PSTD but do not fulfill the criteria for PTSD when ICD‐11 is implemented, will be an important target in the future to address. However, this is not within the scope of this article.

We calculated conditional probabilities for ICD‐11 PTSD and CPTSD for the overall sample and within the ICD‐10 PTSD group, given any traumatic experience. In the overall sample, CPTSD alone was associated with serious harm you caused to someone else and natural disaster. Our results are somewhat different from earlier studies finding that CPTSD occurs more frequently after exposure to traumatic experiences of an interpersonal nature that happened in either childhood or adulthood (24, 35). Natural disaster is seen as a single‐event, non‐interpersonal, non‐prolonged traumatic experience, and in our data was associated with CPTSD. Accumulating evidence (25, 30, 35, 51) suggests that traumatic experiences should be considered a risk factor and that individual vulnerability patterns, event severity, and environmental and protective factors may be relevant to symptom pattern development (34). The association between CPTSD and serious harm you caused to someone else shown in our results indicates that individuals who commit a violent act may be associated with CPTSD. Evidence has presented a relationship between childhood abuse and adult violence (52) and between exposure to traumatic experiences, PTSD, and perpetration (53), which resemble our findings. When we look specifically at those within the ICD‐10 PTSD group, the conditional probability for CPTSD had a stronger association with neglect and natural disaster, and ICD‐11 PTSD alone was associated with exposure to a war‐zone. Our findings do not reflect a clear picture of the conditional probability for ICD‐11 CPTSD vs. PTSD given specific types of traumatic experiences.

Finally, we investigated whether ICD‐11 PTSD and CPTSD could be differentiated in our sample in terms of psychological well‐being and exposure to multiple traumatic experiences. In contrast to other findings (22, 23), we did not find any significant difference in any of these factors. This inconsistency may be related to the nature of our sample. In general, the mean total well‐being score was low (mean = 6.6, SD = 4.5), indicating the level of suffering to be profound in our sample. Our sample consisted of treatment‐seeking psychiatric patients, suffering at the extreme end of symptom severity; consequently, these factors might not be effective predictors for distinguishing the two disorders in such a highly affected psychiatric population. Nevertheless, more evidence from larger samples for each ICD‐11 disorder is warranted within a psychiatric population to further investigate differential factors.

Our study has a number of limitations. We used a relatively small sample from three Danish outpatient clinics. We were unable to estimate the response rate since we could not control whether all consecutively referred patients were asked to participate, although the clinics were instructed to do so. Furthermore, we had low numbers of participants in each ICD‐10 disorder group, which may compromise and under power the statistical comparisons. However, most of our findings are consistent with previous studies investigating trauma and PTSD in a psychiatric population. We were unable to check the self‐reported ICD‐11 diagnoses with diagnostic interviews for potential response bias. To fulfill the criteria of a traumatic experience, we used endorsement of at least one item on the LEC measure instead of the identified worst trauma written by the participant on the ITQ. We encountered low endorsements on some of the specific traumatic experiences in our sample, which may affect the calculation of the specific conditional probabilities and limit the conclusions about specific conditional disorder prevalence. Last, we used ICD‐10 diagnoses from the hospital chart and could not test the legitimacy of these diagnoses with a structured diagnostic interview.

The results of this study show high levels of traumatic experiences in almost all patients in our sample. ICD‐11 CPTSD was shown to be a more common condition than PTSD in our sample of psychiatric treatment‐seeking patients. We found CPTSD to overlap with all of the diagnosed ICD‐10 disorders; affective, anxiety, PTSD, BPD, other personality disorders, adjustment and stress‐reaction, and behavioural and emotional disorders with onset usually occurring in childhood and adolescence; whereas PTSD was found to only overlap with anxiety, PTSD, BPD, adjustment and stress‐reaction, and behavioural and emotional disorders with onset usually occurring in childhood and adolescence. Furthermore, almost one quarter (23%) having an ICD‐10 PTSD diagnosis did not meet criteria for either of the ICD‐11 diagnoses. Similar studies with larger sample sizes that investigate a heterogenetic psychiatric sample are warranted for future research, to investigate the similarity and difference between ICD‐11 PTSD and CPTSD, and differential diagnostics for other psychiatric conditions. Such knowledge is essential for clinical utility, specifically with regard to correct diagnosis where familiarity with differential diagnostics is vital.

## Declaration of interest

The authors report no conflict of interest.

## Data Availability

The data that support the findings of this study are available from the corresponding author upon request.
